# Viral Load Dynamics in Sputum and Nasopharyngeal Swab in Patients with COVID-19

**DOI:** 10.1177/0022034520946251

**Published:** 2020-08-03

**Authors:** R. Liu, S. Yi, J. Zhang, Z. Lv, C. Zhu, Y. Zhang

**Affiliations:** 1 Department of Clinical Laboratory, Renmin Hospital of Wuhan University, Wuhan, China; 2 The State Key Laboratory Breeding Base of Basic Science of Stomatology (Hubei-MOST) and Key Laboratory of Oral Biomedicine Ministry of Education, School and Hospital of Stomatology, Wuhan University, Wuhan, China; 3 State Key Laboratory of Virology, College of Life Sciences, Wuhan University, Wuhan, China

**Keywords:** coronavirus, virus infections, virulence, serial passage, bodily secretions, public health

## Abstract

Coronavirus disease 2019 (COVID-19) has caused a global pandemic associated with substantial morbidity and mortality. Nasopharyngeal swabs and sputum samples are generally collected for serial viral load screening of respiratory contagions, but temporal profiles of these samples are not completely clear in patients with COVID-19. We performed an observational cohort study at Renmin Hospital of Wuhan University, which involved 31 patients with confirmed COVID-19 with or without underlying diseases. We obtained samples from each patient, and serial viral load was measured by real-time quantitative polymerase chain reaction. We found that the viral load in the sputum was inclined to be higher than samples obtained from the nasopharyngeal swab at disease presentation. Moreover, the viral load in the sputum decreased more slowly over time than in the nasopharyngeal group as the disease progressed. Interestingly, even when samples in the nasopharyngeal swab turned negative, it was commonly observed that patients with underlying diseases, especially hypertension and diabetes, remained positive for COVID-19 and required a longer period for the sputum samples to turn negative. These combined findings emphasize the importance of tracking sputum samples even in patients with negative tests from nasopharyngeal swabs, especially for those with underlying conditions. In conclusion, this work reinforces the importance of sputum samples for SARS-CoV-2 detection to minimize transmission of COVID-19 within the community.

## Introduction

Coronavirus disease 2019 (COVID-19), a novel viral pneumonia, was first widely reported in Wuhan, the capital city of Hubei province in China (Li et al. 2020; Zhu et al. 2020). The virus has been identified as a novel species, namely severe acute respiratory syndrome coronavirus 2 (SARS-CoV-2), which has similarity to the SARS-CoV pathogen (Zou et al. 2020). Although the mortality rate from SARS-CoV infection was higher than that of COVID-19, SARS-CoV-2 can spread much faster and has led to a global pandemic (Rainer et al. 2007; Li et al. 2020; Wang, Hu, et al. 2020). The World Health Organization (WHO) declared COVID-19 an international public health emergency on January 30, 2020 (Guan et al. 2020). By July 7, 2020, the United States had reported the most deaths, with 132,979. Brazil, Britain, and Italy followed with nearly 96,000, 45,000, and 35,000 deaths, respectively. Epidemiologists and governments all over the world are striving to analyze cases that may help ascertain the defining clinical characteristics.

Given the rapid spread and respiratory symptoms of COVID-19, clinicians and scientists have determined that the virus spreads mainly through dispersal of droplets, generated when patients talk, cough, and sneeze (Guan et al. 2020; Lo et al. 2020; Wang, Hu, et al. 2020). In terms of respiratory infections, successive sampling of nasopharyngeal swabs, throat swabs, and sputum (secreted from the oropharynx and oral cavity) is usually used for viral load monitoring (Lin et al. 2020; Lo et al. 2020; Zou et al. 2020). Unlike SARS-CoV, SARS-CoV-2 (inversely related to the cycle threshold [Ct] value) peaks soon after symptom onset, with higher viral loads in the nose than in the throat (Pan et al. 2020; Wu et al. 2020; Yip et al. 2020). But sputum samples are more commonly collected, and the relationship between the viral load of the nasopharyngeal samples and the sputum samples is still unclear. With the control of the disease and the intervention of antiviral drugs, the viral load can be reduced, but the time required for the nasopharyngeal swab and sputum to turn negative varies. However, the viral load dynamics and characteristics in nasopharyngeal secretions and sputum samples during course of the COVID-19 disease remain unclear.

It has been reported that patients with systemic diseases such as high blood pressure, asthma, and diabetes are more likely to be infected with the virus (Chen, Dai, et al. 2020; Deng et al. 2020; Xie et al. 2020). Moreover, patients with underlying diseases have lower immunity and increased blood sugar facilitating bacterial infection, which extends the course of the disease (Wang, Hu, et al. 2020). Therefore, in a retrospective study of the disease, it is important to distinguish the duration of the disease and how it may affect different patient populations.

We systematically analyzed the viral load dynamics by Ct value in >300 samples collected from 31 patients with and without underlying medical conditions, after confirmation of COVID-19. Two test sampling collecting methods were used for sputum and nasopharyngeal swab samples. Both sampling methods were investigated for differences in the required time for patient samples to turn negative.

## Materials and Methods

### Study Design and Patients

This was a retrospective cohort study of patients with laboratory-confirmed COVID-19 who were admitted consecutively to Renmin Hospital, Wuhan University. All patients were diagnosed as having SARS-CoV-2 infection according to the WHO interim guidance. Patients were divided into 2 groups: 16 adults with underlying diseases and 15 adults without underlying diseases, all of whom were admitted to the hospital. The study conformed to the ethical guidelines of the 1975 Declaration of Helsinki and was approved by the ethics committee of the Renmin Hospital, Wuhan University (No. WDRY2020-K066).

### Sample Processing

The sputum and nasopharyngeal swab samples were collected according to the guidelines for collection. Sputum samples were collected from the respiratory tract. The patient was asked to rinse his or her mouth with water and then expectorate deep-cough sputum directly into a sterile container. Nasopharyngeal swabs were collected with a commercially available plastic rod swab with a polypropylene fiber head (Biotron). The swab was gently passed up the nostril to the posterior wall of the nasopharynx, held for approximately 3 s, rotated 2 or 3 times, withdrawn gently, and placed in 3 mL of virus transfer medium.

### Inclusion Criteria

During whole treatment, patients diagnosed with SARS-CoV-2 infection via sputum and nasopharyngeal swab samples according to the WHO interim guidance were included in this study. Patients with former or current diabetes, cardiovascular disease, malignant tumor, and so on were identified as the underlying disease group.

### Exclusion Criteria

Patients diagnosed with SARS-CoV-2 infection via a single method of sputum or nasopharyngeal swab samples or with incomplete test results recorded were excluded from the study.

### Viral Extraction and Real-time Quantitative Polymerase Chain Reaction

The extraction procedure was performed via an extraction kit (Health Gene Technologies) and nucleic acid extractor (TANBead) according to the manufacturers’ protocol. Samples were tested by real-time quantitative polymerase chain reaction (RT-qPCR) amplification for SARS-CoV-2 open reading frame (ORF) 1ab and nucleocapsid protein (NP) gene fragments per the double nucleic acid detection kit (BioGerm), following WHO guidelines (Corman et al. 2020). Samples with Ct values were considered positive for SARS-CoV-2 RNA, while samples with undetectable Ct values were consider negative.

### Data Collection and Statistical Analysis

The research team of Wuhan University Renmin Hospital analyzed the medical records of patients. After analysis, the distributions of viral load in sputum and nasopharyngeal swab samples and all data were of normal distribution. For statistical analysis in the study, unpaired Student’s *t* test was used to evaluate the correlation between viral amount in sputum and nasopharyngeal swab samples and the correlation between patients with and without underlying diseases (Prism 8.0, GraphPad; SPSS 25.0, IBM).

## Results

### Correlation between Viral Load in Sputum and Nasopharyngeal Swab Samples

**Figure 1.**
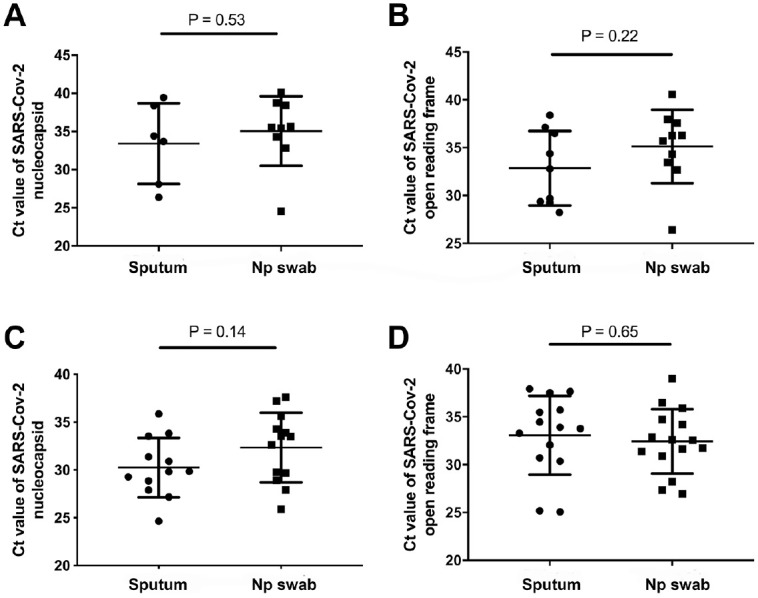
Detection of severe acute respiratory syndrome coronavirus 2 (SARS-CoV-2) in patients with and without underlying diseases in different sample types. Comparison of SARS-CoV-2 Ct values in sputum and nasopharyngeal swab samples without underlying diseases: (**A**) NP and (**B**) ORF. Comparison of SARS-CoV-2 NP Ct values in sputum and nasopharyngeal swab samples with underlying diseases: (**C**) NP and (**D**) ORF. Values are presented as mean ± SD. Ct, cycle threshold; NP, nucleocapsid protein; ORF, open reading frame 1ab.

The demographic and clinical information of the 16 patients with underlying diseases and 15 patients without underlying diseases at the initial day are summarized in Appendix Tables 1 and 2. Viral RNA was extracted from sputum and nasopharyngeal swabs and subjected to an RT-qPCR assay. The sampling day of these patients varies from day 1 to day 13 after admittance to the hospital. In patients without underlying diseases, the Ct values were 28.23 to 38.39 (mean, 32.85) for SARS-CoV-2 ORF from the sputum samples and 26.37 to 39.45 (mean, 33.4) for SARS-CoV-2 NP in sputum samples. SARS-CoV-2 RNA was also detected in nasopharyngeal swab samples from all 15 available specimens. The Ct values were 26.41 to 40.58 (mean, 35.13) for SARS-CoV-2 ORF in nasopharyngeal swab samples and 24.55 to 40.11 (mean, 35.05) for SARS-CoV-2 NP in nasopharyngeal swab samples. For the 16 patients with underlying diseases, sputum and nasopharyngeal swab samples were also evaluated by RT-qPCR and are listed in Appendix Table 2. The Ct values were 25.17 to 37.64 (mean, 33.07) for SARS-CoV-2019 ORF in sputum samples and 24.65 to 35.86 (mean, 30.25) for SARS-CoV-2 NP in sputum samples. For the nasopharyngeal swab samples, the Ct values were 26.93 to 36.46 (mean, 32.43) for SARS-CoV-2 ORF and 25.88 to 37.63 (mean, 32.34) for SARS-CoV-2 NP. In both groups, the Ct values of SARS-CoV-2 ORF and NP in sputum samples were smaller than in the nasopharyngeal swab samples, indicating higher viral load in the sputum samples (Fig. 1).

### Correlation of Viral Duration in Different Sample Types

**Figure 2.**
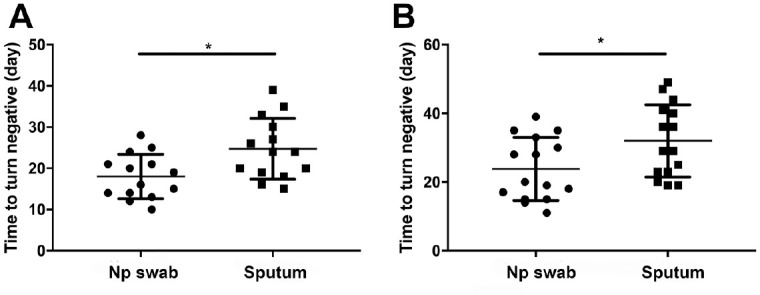
Comparison of the time required for samples to turn negative in the sputum and nasopharyngeal swab sample groups: (**A**) patients without underlying diseases and (**B**) patients with underlying diseases. Values are presented as mean ± SD. *P < 0.05.

To demonstrate the duration of SARS-CoV-2 in sputum and nasopharyngeal swab samples, we collected demographic and clinical information of 15 patients without underlying diseases and 16 patients with underlying diseases and tested them over time to determine when the viral load turned negative. In patients without underlying diseases, the sampling day of these patients varied from day 10 to day 28 after admission to the hospital. At these points, sputum and nasopharyngeal swab samples were collected and tested and are summarized in Appendix Tables 3 and 4. As demonstrated, the Ct values were 25.29 to 38.96 (mean, 34.32) for SARS-CoV-2 ORF in sputum samples and 26.13 to 41.16 (mean, 34.25) for SARS-CoV-2 NP, while most nasopharyngeal swab samples exhibited negative results. For the 16 patients with underlying diseases, the Ct values were 22.19 to 39.91 (mean, 34.75) for SARS-CoV-2 ORF in sputum samples and 22.46 to 41.8 (mean, 35.02) for SARS-CoV-2 NP, while most nasopharyngeal swab samples also exhibited negative results. When compared with the nasopharyngeal swab samples, the sputum samples needed a delayed duration to turn negative (Fig. 2).

### Correlation Between Viral Duration and Underlying Diseases

**Figure 3.**
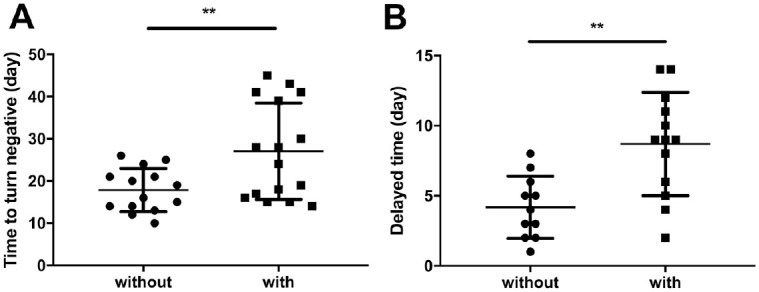
Association between underlying diseases and duration of severe acute respiratory syndrome coronavirus-2 infection. (**A**) The duration of nasopharyngeal swab samples turning negative in patients with and without underlying diseases. (**B**) The delayed time required for samples to turn negative in the sputum samples in patients with and without underlying diseases. Values are presented as mean ± SD. **P < 0.005.

To investigate the correlation between viral duration and underlying diseases, we collected and compared the demographic and clinical symptoms of patients with and without underlying diseases (Appendix Table 5). In patients with underlying diseases, the viral duration of the nasopharyngeal swab samples was longer than patients without underlying diseases (Fig. 3A). In addition, there was a delayed duration for when the viral sample turned negative in the sputum group without underlying diseases (Fig. 3B).

## Discussion

SARS-CoV-2 can accumulate in the nasopharynx, oropharynx, and oral cavity, which contributes to its disease transmission through oral droplets (To, Tsang, Leung, et al. 2020). In the present study, we surveyed the different temporal viral load profiles of 31 inpatients with COVID-19 and analyzed the relationship between sample type and viral load in nasopharyngeal and sputum samples of 2 patient populations. The length of time required for samples to turn negative was also determined and analyzed. The results suggest that viral load in the sputum is higher than that in the nasopharyngeal swab at disease presentation. Moreover, viral load in the sputum samples decreased more slowly than in the nasopharyngeal swab samples as the disease progressed. Although the nasopharyngeal swab samples turned negative, especially in patients with an underlying disease, such as hypertension and diabetes, a longer time was required for sputum samples to turn negative.

Since the outbreak of the COVID-19 disease, clinicians have successively tested various samples of patients and used RT-qPCR to detect the viral load and virus proliferation rate in the body (Yip et al. 2020; Zou et al. 2020). In addition to the nasopharyngeal swabs and sputum sample testing described in this article, other samples tested included feces, anal swabs, blood, and urine (To, Tsang, Leung, et al. 2020; Zheng et al. 2020). Additionally, some hospitals would test serum anti-SARS-CoV-2 antibodies to track their immune status (Wolfel et al. 2020). However, in the screening of a large number of suspected patients and in the asymptomatic population, nasopharyngeal swabs and sputum are still heavily used by medical staff and patients because of their ease and convenience (Goh et al. 2017; He et al. 2020; To, Tsang, Chik-Yan, et al. 2020; Yip et al. 2020). Therefore, in-depth analysis and understanding of the disease from nasopharyngeal swabs and saliva sputum are necessary. Consistent with recent studies, we reported that a high viral load on the onset of COVID-19 was detected and declined steadily (To, Tsang, Leung, et al. 2020). It was shown that SARS-CoV-2 RNA could be detected in nasopharyngeal swabs and sputum samples at a mean sampling time of 6.7 d (range, 1 to 13 d) in patients without underlying disease, compared with 4.8 d (range, 1 to 8 d) in patients with underlying disease. This suggests that the virus spreads and replicates faster in the patient population with underlying diseases. Moreover, we found that the viral load of the sputum sample was slightly higher than that in the nasopharyngeal swab at the beginning of the disease, regardless of underlying disease, indicating that lower respiratory tract secretions have a higher virus transmission capacity.

It has also been recognized that the virus shedding time is different in different parts of the respiratory tract (He et al. 2020; Wolfel et al. 2020; Zheng et al. 2020). It was shown that the peak of SARS-CoV-2 in the upper respiratory tract specimens appears at the early stage of the disease (Guan et al. 2020). Yet another study reported that the virus shedding time is longer in lower respiratory tract secretions and that the maximum viral loads appear about 2 wk after the onset of symptoms (Zheng et al. 2020). In the current study, we consistently found longer SARS-CoV-2 detection in the sputum samples than in the nasopharyngeal swab samples collected from patients with or without underlying diseases, delayed by a mean 8.3 and 4.1 d, respectively. The findings were also similar to the latest study by Wang, Zhang, et al. (2020). But the delayed shedding duration of sputum samples that they counted was longer than the one in this article, which may be attributed to the different included patient population. The different expression levels of ACE2 (angiotensin converting enzyme 2), the putative cell entry receptor of SARS-CoV-2, in these regions may partly explain this phenomenon. Meanwhile, the lower respiratory tract communicates less with the outside world than the upper respiratory tract, which may exacerbate viral retention. In patients with pulmonary disease, even if the clinical symptoms are cured, there is still a certain amount of viral load, leading to prolonged shedding of the virus in lower respiratory secretions and sputum (Huang et al. 2020; Liu et al. 2020; Wolfel et al. 2020). These findings are vital for resultful management and prevention of the epidemic in hospital and community populations, as it remains impossible to ensure that there is no virus load in the body following a negative nasopharyngeal swab test and that patients still need to be strictly managed accordingly (Wolfel et al. 2020). Furthermore, patients with underlying diseases should be treated more closely due to their faster disease onset and longer course of disease transmission potential.

The health status of patients before the infection of the virus affects the body’s ability to respond to it and causes different severity of disease (Chen, Wu, et al. 2020; Zhou et al. 2020). It was shown that comorbidities existed in almost half of inpatients, with hypertension being the most common, followed by diabetes and coronary heart disease (Deng and Peng 2020). Therefore, the existence of underlying diseases such as hypertension and diabetes is one of the important factors affecting the prognosis of patients (Deng et al. 2020). We found that in the group with the underlying diseases, the nasopharyngeal swab and sputum groups took longer to turn negative than the group without the underlying diseases, with the sputum samples remaining positive the longest, consistent with other research (Wang, Zhang, et al. 2020). Meanwhile, this study pointed out that elderly patients might have prolonged viral shedding duration, and it suggested that diabetes was associated with the detection of virus RNA from nasopharyngeal swab and sputum specimens. Based on these observations, it may be advisable to make a powerful and reasonable treatment plan and provide antiviral treatment for patients, under the premise of controlling basic conditions (Guan et al. 2020).

Our study has limitations. First, viral load can be affected by several factors. We reported only the Ct values (not exact viral loads). So the study of viral load cannot perfectly reflect the exact amount of the virus in the body (To, Tsang, Leung, et al. 2020). Second, RT-qPCR cannot guarantee the detection of viable viruses (Zheng et al. 2020; Zou et al. 2020). Third, the low sample size did not allow for more subgroups, such as age, sex, severity of disease, and so on (Chan et al. 2020). When viral shedding time is assessed, it may lead to an unbalanced distribution of other factors. Finally, we focused on the respiratory tract samples but ignored the blood biochemical indicators, and we did not integrate all the laboratory results and course of disease for analysis.

COVID-19 is a public health emergency with many remaining unknowns. The current study has demonstrated the viral shedding pattern in nasopharynx and oropharynx secretions from patients with or without underlying conditions, which may assist in determining the epidemiologic dynamics of the virus and in updating monitoring practices. These findings indicate that decreasing viral loads, specifically in sputum, would be conducive to prevent the spread of the virus since these samples contain higher viral loads for longer periods. Pathogenic biological and immunologic studies remain needed to deeply understand SARS-CoV-2 infection characteristics at the earliest convenience to curb transmission of the disease.

## Author Contributions

R. Liu, contributed to conception and design, drafted the manuscript; S. Yi, J. Zhang, contributed to data acquisition, analysis, or interpretation, drafted the manuscript; Z. Lv, contributed to data acquisition, analysis, or interpretation, critically revised the manuscript; C. Zhu, Y. Zhang, contributed to conception and design, critically revised the manuscript. All authors gave final approval and agree to be accountable for all aspects of the work.

## Supplemental Material

DS_10.1177_0022034520946251 – Supplemental material for Viral Load Dynamics in Sputum and Nasopharyngeal Swab in Patients with COVID-19 Click here for additional data file. Supplemental material, DS_10.1177_0022034520946251 for Viral Load Dynamics in Sputum and Nasopharyngeal Swab in Patients with COVID-19 by R. Liu, S. Yi, J. Zhang, Z. Lv, C. Zhu and Y. Zhang in Journal of Dental Research

## References

[bibr1-0022034520946251] Chan JF Yuan S Kok KH To KK Chu H Yang J Xing F Liu J Yip CC Poon RW, et al 2020 A familial cluster of pneumonia associated with the 2019 novel coronavirus indicating person-to-person transmission: a study of a family cluster. Lancet. 395(10223):514–523. 31986261 10.1016/S0140-6736(20)30154-9 PMC7159286

[bibr2-0022034520946251] Chen T Dai Z Mo P Li X Ma Z Song S Chen X Luo M Liang K Gao S, et al 2020 Clinical characteristics and outcomes of older patients with coronavirus disease 2019 (COVID-19) in Wuhan, China (2019): a single-centered, retrospective study. J Gerontol A Biol Sci Med Sci [epub ahead of print 11 Apr 2020] in press. doi:10.1093/gerona/glaa089 PMC7184388 32279081

[bibr3-0022034520946251] Chen T Wu D Chen H Yan W Yang D Chen G Ma K Xu D Yu H Wang H, et al 2020 Clinical characteristics of 113 deceased patients with coronavirus disease 2019: retrospective study. BMJ. 368:m1091. 32217556 10.1136/bmj.m1091 PMC7190011

[bibr4-0022034520946251] Corman VM Landt O Kaiser M Molenkamp R Meijer A Chu DK Bleicker T Brunink S Schneider J Schmidt ML, et al 2020 Detection of 2019 novel coronavirus (2019-nCoV) by real-time RT-PCR. Euro Surveill. 25(3):2000045. 10.2807/1560-7917.ES.2020.25.3.2000045 PMC6988269 31992387

[bibr5-0022034520946251] Deng SQ Peng HJ 2020 Characteristics of and public health responses to the coronavirus disease 2019 outbreak in China. J Clin Med. 9(2):575. 10.3390/jcm9020575 PMC7074453 32093211

[bibr6-0022034520946251] Deng Y Liu W Liu K Fang YY Shang J Zhou L Wang K Leng F Wei S Chen L, et al 2020 Clinical characteristics of fatal and recovered cases of coronavirus disease 2019 (COVID-19) in Wuhan, China: a retrospective study. Chin Med J (Engl). 133(11):1261–1267. 32209890 10.1097/CM9.0000000000000824 PMC7289311

[bibr7-0022034520946251] Goh EH Jiang L Hsu JP Tan LWL Lim WY Phoon MC Leo YS Barr IG Chow VTK Lee VJ, et al 2017 Epidemiology and relative severity of influenza subtypes in Singapore in the post-pandemic period from 2009 to 2010. Clin Infect Dis. 65(11):1905–1913. 29028950 10.1093/cid/cix694 PMC5850443

[bibr8-0022034520946251] Guan WJ Ni ZY Hu Y Liang WH Ou CQ He JX Liu L Shan H Lei CL Hui DSC, et al 2020 Clinical characteristics of coronavirus disease 2019 in China. N Engl J Med. 382(18):1708–1720. 32109013 10.1056/NEJMoa2002032 PMC7092819

[bibr9-0022034520946251] He X Lau EHY Wu P Deng X Wang J Hao X Lau YC Wong JY Guan Y Tan X, et al 2020 Temporal dynamics in viral shedding and transmissibility of COVID-19. Nat Med. 26(5):672–675. 32296168 10.1038/s41591-020-0869-5

[bibr10-0022034520946251] Huang Y Chen S Yang Z Guan W Liu D Lin Z Zhang Y Xu Z Liu X Li Y 2020 SARS-CoV-2 viral load in clinical samples from critically ill patients. Am J Respir Crit Care Med. 201(11):1435–1438. 32293905 10.1164/rccm.202003-0572LE PMC7258645

[bibr11-0022034520946251] Li Q Guan X Wu P Wang X Zhou L Tong Y Ren R Leung KSM Lau EHY Wong JY, et al 2020 Early transmission dynamics in Wuhan, China, of novel coronavirus-infected pneumonia. N Engl J Med. 382(13):1199–1207. 31995857 10.1056/NEJMoa2001316 PMC7121484

[bibr12-0022034520946251] Lin C Xiang J Yan M Li H Huang S Shen C 2020 Comparison of throat swabs and sputum specimens for viral nucleic acid detection in 52 cases of novel coronavirus (SARS-CoV-2)-infected pneumonia (COVID-19). Clin Chem Lab Med. 58(7):1089–1094. 32301745 10.1515/cclm-2020-0187

[bibr13-0022034520946251] Liu WD Chang SY Wang JT Tsai MJ Hung CC Hsu CL Chang SC 2020 Prolonged virus shedding even after seroconversion in a patient with COVID-19. J Infect. 81(2):318–356. 10.1016/j.jinf.2020.03.063 PMC7151379 32283147

[bibr14-0022034520946251] Lo IL Lio CF Cheong HH Lei CI Cheong TH Zhong X Tian Y Sin NN 2020 Evaluation of SARS-CoV-2 RNA shedding in clinical specimens and clinical characteristics of 10 patients with COVID-19 in Macau. Int J Biol Sci. 16(10):1698–1707. 32226287 10.7150/ijbs.45357 PMC7098032

[bibr15-0022034520946251] Pan Y Guan H Zhou S Wang Y Li Q Zhu T Hu Q Xia L 2020 Initial CT findings and temporal changes in patients with the novel coronavirus pneumonia (2019-nCoV): a study of 63 patients in Wuhan, China. Eur Radiol. 30(6):3306–3309. 32055945 10.1007/s00330-020-06731-x PMC7087663

[bibr16-0022034520946251] Rainer TH Lee N Ip M Galvani AP Antonio GE Wong KT Chan DP Ng AW Shing KK Chau SS, et al 2007 Features discriminating SARS from other severe viral respiratory tract infections. Eur J Clin Microbiol Infect Dis. 26(2):121–129. 17219094 10.1007/s10096-006-0246-4 PMC7088160

[bibr17-0022034520946251] To KK Tsang OT Chik-Yan Yip C Chan KH Wu TC Chan JMC Leung WS Chik TS Choi CY Kandamby DH, et al 2020 Consistent detection of 2019 novel coronavirus in saliva. Clin Infect Dis. 71(15):841–843. 32047895 10.1093/cid/ciaa149 PMC7108139

[bibr18-0022034520946251] To KK Tsang OT Leung WS Tam AR Wu TC Lung DC Yip CC Cai JP Chan JM Chik TS, et al 2020 Temporal profiles of viral load in posterior oropharyngeal saliva samples and serum antibody responses during infection by SARS-CoV-2: an observational cohort study. Lancet Infect Dis. 20(5):565–574. 32213337 10.1016/S1473-3099(20)30196-1 PMC7158907

[bibr19-0022034520946251] Wang D Hu B Hu C Zhu F Liu X Zhang J Wang B Xiang H Cheng Z Xiong Y, et al 2020 Clinical characteristics of 138 hospitalized patients with 2019 novel coronavirus-infected pneumonia in Wuhan, China. JAMA. 323(11):1061–1069. 32031570 10.1001/jama.2020.1585 PMC7042881

[bibr20-0022034520946251] Wang K Zhang X Sun J Ye J Wang F Hua J Zhang H Shi T Li Q Wu X 2020 Differences of SARS-CoV-2 shedding duration in sputum and nasopharyngeal swab specimens among adult inpatients with COVID-19. Chest [epub ahead of print 20 Jun 2020] in press. doi:10.1016/j.chest.2020.06.015 PMC7305751 32569635

[bibr21-0022034520946251] Wolfel R Corman VM Guggemos W Seilmaier M Zange S Muller MA Niemeyer D Jones TC Vollmar P Rothe C, et al 2020 Virological assessment of hospitalized patients with COVID-2019. Nature. 581(7809):465–469. 32235945 10.1038/s41586-020-2196-x

[bibr22-0022034520946251] Wu J Liu J Li S Peng Z Xiao Z Wang X Yan R Luo J 2020 Detection and analysis of nucleic acid in various biological samples of COVID-19 patients. Travel Med Infect Dis [epub ahead of print 18 Apr 2020] in press. doi:10.1016/j.tmaid.2020.101673 PMC7165102 32311437

[bibr23-0022034520946251] Xie J Tong Z Guan X Du B Qiu H 2020 Clinical characteristics of patients who died of coronavirus disease 2019 in China. JAMA Netw Open. 3(4):e205619. 32275319 10.1001/jamanetworkopen.2020.5619 PMC7148440

[bibr24-0022034520946251] Yip CC Ho CC Chan JF To KK Chan HS Wong SC Leung KH Fung AY Ng AC Zou Z, et al 2020 Development of a novel, genome subtraction-derived, SARS-CoV-2-specific COVID-19-nsp2 real-time RT-PCR assay and its evaluation using clinical specimens. Int J Mol Sci. 21(7):2574. 10.3390/ijms21072574 PMC7177594 32276333

[bibr25-0022034520946251] Zheng S Fan J Yu F Feng B Lou B Zou Q Xie G Lin S Wang R Yang X, et al 2020 Viral load dynamics and disease severity in patients infected with SARS-CoV-2 in Zhejiang province, China, January–March 2020: retrospective cohort study. BMJ. 369:m1443. 32317267 10.1136/bmj.m1443 PMC7190077

[bibr26-0022034520946251] Zhou F Yu T Du R Fan G Liu Y Liu Z Xiang J Wang Y Song B Gu X, et al 2020 Clinical course and risk factors for mortality of adult inpatients with COVID-19 in Wuhan, China: a retrospective cohort study. Lancet. 395(10229):1054–1062. 32171076 10.1016/S0140-6736(20)30566-3 PMC7270627

[bibr27-0022034520946251] Zhu N Zhang D Wang W Li X Yang B Song J Zhao X Huang B Shi W Lu R, et al 2020 A novel coronavirus from patients with pneumonia in China, 2019. N Engl J Med. 382(8):727–733. 31978945 10.1056/NEJMoa2001017 PMC7092803

[bibr28-0022034520946251] Zou L Ruan F Huang M Liang L Huang H Hong Z Yu J Kang M Song Y Xia J, et al 2020 SARS-CoV-2 viral load in upper respiratory specimens of infected patients. N Engl J Med. 382(12):1177–1179. 32074444 10.1056/NEJMc2001737 PMC7121626

