# Supplementary material for: Viral Load Dynamics in Sputum and Nasopharyngeal Swab in Patients with COVID-19
Source: J Dent Res. 2020 Aug 3;99(11):1239–44. doi: 10.1177/0022034520946251 (PMC7399563; doi:10.1177/0022034520946251)
Supplement: DS_10.1177_0022034520946251 – Supplemental material for Viral Load Dynamics in Sputum and Nasopharyngeal Swab in Patients with COVID-19 [file DS_10.1177_0022034520946251.pdf]

Viral load dynamics in sputum and nasopharyngeal swab in COVID-19 patients

Rui Liu[1#], Siqi Yi[2#], Jinglun Zhang[2#], Zhihua Lv[1#], Chengliang Zhu[1*], Yufeng Zhang[2*]

[1] Department of Clinical Laboratory, Renmin Hospital of Wuhan University, Wuhan 430060, Hubei, China;

[2] The State Key Laboratory Breeding Base of Basic Science of Stomatology (Hubei-MOST) & Key Laboratory of Oral Biomedicine Ministry of Education, School & Hospital of Stomatology, Wuhan University, Wuhan 430079, China;

[#]These authors contributed equally to this study

Supplemental Table 1

Patient ID	Age (y)	Sex	Sampling day	Clinical information at sampling day				Sputum (Ct Value)	Nasopharyngeal swab (Ct Value)
				Fever	Cough	Dyspnea	Chest CT		
1	59	M	D3	Yes	Yes	Yes	Yes	NA	34.32(ORF)/38.72(NP)
2	47	M	D4	No	Yes	No	Yes	32.79(ORF)	NA
3	55	M	D11	No	No	No	Yes	36.47(ORF)/39.45(NP)	36.3(ORF)/35.53(NP)
4	52	M	D14	No	No	No	Yes	Positive(NA)	Negative
5	56	F	D7	No	No	No	Yes	28.23(ORF)	Negative
6	79	F	D1	Yes	Yes	Yes	Yes	38.39(ORF)/34.4(NP)	40.58(ORF)/38.44(NP)
7	77	M	D4	No	Yes	No	Yes	34.38(ORF)/33.68(NP)	36.28(ORF)/35.44(NP)
8	46	F	D10	Yes	Yes	No	Yes	29.35(ORF)/28.1(NP)	33.47(ORF)/32.8(NP)
9	45	M	D1	No	Yes	No	Yes	29.7(ORF)/26.37(NP)	26.41(ORF)/24.55(NP)
10	67	M	D13	No	No	No	Yes	37.13(ORF)/38.39(NP)	37.58(ORF)
11	63	F	D5	No	Yes	No	Yes	NA	37.96(ORF)/35.63(NP)
12	57	F	D7	No	Yes	No	Yes	NA	35.67(ORF)
13	66	F	D6	No	No	Yes	Yes	29.23(ORF)	NA
14	65	M	D7	No	Yes	Yes	Yes	NA	32.69(ORF)/34.25(NP)
15	71	M	D7	No	Yes	No	Yes	NA	40.11(NP)

Supplemental Table 1. Personal and clinical information of patients without underlying diseases at initial days.

Supplemental Table 2

Patient ID	Age (y)	Sex	Sampling day	Clinical information at sampling day: Fever	Cough	Dyspnea	Chest CT	Past history	Sputum (Ct Value)	Nasopharyngeal swab (Ct Value)
1	52	M	D2	Yes	No	No	Yes	Steatohepatitis	35.7(ORF)/30.91(NP)	32.56(ORF)/34.27(NP)
2	51	F	D3	No	No	No	Yes	Sinus carcinoma	30.36(ORF)	30.87(ORF)/29.69(NP)
3	64	F	D8	Yes	Yes	No	Yes	HBP DM CHD	30.71(ORF)/29.27(NP)	28.23(ORF)/37.63(NP)
4	72	M	D2	No	No	No	Yes	DM	Positive(NA)	34.73(ORF)/33.88(NP)
5	56	F	D4	Yes	Yes	Yes	Yes	HBP	25.05(ORF)/29.86(NP)	35.91(ORF)/37.22(NP)
6	67	F	D8	Yes	Yes	No	Yes	HBP DM	35.48(ORF)/34.73(NP)	39.01(ORF)/33.53(NP)
7	72	M	D6	Yes	Yes	Yes	Yes	DM	37.92(ORF)/35.91(NP)	34.21(ORF)/32.61(NP)
8	51	M	D4	No	No	Yes	Yes	HBP DM	33.78(ORF)/39.01(NP)	32.62(ORF)/35.64(NP)
9	54	M	D7	Yes	Yes	Yes	Yes	HBP DM	37.64(ORF)/29.83(NP)	36.46(ORF)/27.9(NP)
10	66	F	D6	Yes	Yes	No	Yes	HBP	33.27(ORF)/27.9(NP)	27.32(ORF)
11	65	F	D3	Yes	Yes	No	Yes	HBP DM	NA	31.38(ORF)/33.48(NP)
12	49	F	D8	Yes	No	Yes	Yes	HBP	37.5(ORF)/31.38(NP)	32.85(ORF)
13	48	M	D6	Yes	Yes	No	Yes	HBP HLM DM	34.45(ORF)	31.62(ORF)/28.96(NP)
14	69	M	D1	Yes	No	No	Yes	HBP	33.91(ORF)/33.83(NP)	26.93(ORF)/25.88(NP)
15	75	M	D4	No	Yes	No	Yes	HBP DM CHD	25.17(ORF)/28.85(NP)	31.7(ORF)/29.76(NP)
16	74	F	D5	Yes	Yes	Yes	Yes	HBP	32.06(ORF)/27.17(NP)	NA

Supplemental Table 2. Personal and clinical information of patients with underlying diseases at initial days.

Supplemental Table 3

Patient ID	Age (y)	Sex	Sampling day	Clinical information at sampling day				Sputum (Ct Value)	Nasopharyngeal swab (Ct Value)
				Fever	Cough	Dyspnea	Chest CT		
1	59	M	D28	No	No	No	Yes	34.31(ORF)	Negative
2	47	M	D14	No	Yes	No	Yes	33.97(ORF)	Negative
3	55	M	D19	No	No	No	Yes	36.39(ORF)/39.68(NP)	Negative
4	52	M	D24	No	No	No	Yes	Positive(NA)	Negative
5	56	F	D12	No	No	No	Yes	35.62(ORF)/32.97(NP)	Negative
6	79	F	D11	NA	NA	NA	NA	NA	NA
7	77	M	D16	No	No	No	Yes	NA	Negative
8	46	F	D20	No	No	No	Yes	27.35(ORF)/26.13(NP)	Negative
9	45	M	D13	No	No	No	Yes	25.29(ORF)/33.58(NP)	Negative
10	67	M	D15	No	No	No	Yes	36.51(ORF)/39.26(NP)	Negative
11	63	F	D21	No	Yes	No	Yes	38.31(ORF)	Negative
12	57	F	D14	No	Yes	Yes	Yes	38.96(ORF)/41.16(NP)	Negative
13	66	F	D21	No	No	No	Yes	36.86(NP)	Negative
14	65	M	D25	No	No	No	Yes	29.38(ORF)/29.2(NP)	Negative
15	71	M	D10	No	Yes	No	Yes	38.89(ORF)/29.42(NP)	Negative

Supplemental Table 3. Personal and clinical information of patients without underlying diseases at nasopharyngeal swab samples turning negative point.

Supplemental Table 4

Patient ID	Age (y)	Sex	Sampling day	Clinical information at sampling day				Past history	Sputum (Ct Value)	Nasopharyngeal swab (Ct Value)
				Fever	Cough	Dyspnea	Chest CT			
1	52	M	D20	No	Yes	No	No	Steatohepatitis	37.8(ORF)/38.91(NP)	Negative
2	51	F	D35	No	No	No	No	Sinus carcinoma	22.19(ORF)/22.46(NP)	Negative
3	64	F	D15	No	No	No	No	HBP DM CHD	NA	NA
4	72	M	D19	No	No	No	No	DM	Positive(NA)	Negative
5	56	F	D28	No	No	No	No	HBP	30.71(ORF)/27.6(NP)	Negative
6	67	F	D30	No	No	No	No	HBP DM	34.3(ORF)/32.53(NP)	Negative
7	72	M	D39	No	No	No	No	DM	30.24(ORF)/32.66(NP)	Negative
8	51	M	D33	No	No	No	No	HBP DM	40.73(ORF)	Negative
9	54	M	D11	No	Yes	No	No	HBP DM	38.41(ORF)/40.87(NP)	Negative
10	66	F	D17	No	No	Yes	No	HBP	37.64(ORF)	Negative
11	65	F	D41	No	No	No	No	HBP DM	NA	Negative
12	49	F	D18	No	No	Yes	No	HBP	36.6(ORF)/40.18(NP)	Negative
13	48	M	D14	No	Yes	Yes	No	HBP HLM DM	34.45(ORF)/37.94(NP)	Negative
14	69	M	D15	No	No	No	No	HBP	33.92(ORF)	Negative
15	75	M	D28	Yes	No	No	No	HBP DM CHD	34.84(ORF)/35.2(NP)	Negative
16	74	F	D35	No	Yes	No	No	HBP	39.91(ORF)/41.8(NP)	NA

Supplemental Table 4. Personal and clinical information of patients with underlying diseases at nasopharyngeal swab samples turning negative point.

Supplemental Table 5

Without underlying diseases					With underlying diseases					
Patient ID	Age (y)	Sex	Sampling day	Sputum (Ct Value)	Patient ID	Age (y)	Sex	Sampling day	Past history	Sputum (Ct Value)
1	59	M	D32	Negative	1	52	M	D29	Steatohepatitis	Negative
2	47	M	D16	Negative	2	51	F	D49	Sinus carcinoma	Negative
3	55	M	D26	Negative	3	64	F	NA	HBP DM CHD	NA
4	52	M	D29	Negative	4	72	M	D23	DM	Negative
5	56	F	D15	Negative	5	56	F	D40	HBP	Negative
6	79	F	NA	NA	6	67	F	D36	HBP DM	Negative
7	77	M	D18	Negative	7	72	M	D41	DM	Negative
8	46	F	NA	NA	8	51	M	D47	HBP DM	Negative
9	45	M	D16	Negative	9	54	M	D20	HBP DM	Negative
10	67	M	D23	Negative	10	66	F	NA	HBP	NA
11	63	F	NA	NA	11	65	F	NA	HBP DM	NA
12	57	F	D19	Negative	12	49	F	D29	HBP	Negative
13	66	F	NA	NA	13	48	M	D19	HBP HLM DM	Negative
14	65	M	D26	Negative	14	69	M	D25	HBP	Negative
15	71	M	D16	Negative	15	75	M	D36	HBP DM CHD	Negative
					16	74	F	D44	HBP	Negative

Supplemental Table 5. The delayed turning negative times of phlegm in patients with and without underlying diseases.
